# Which rehabilitation initiatives can effectively improve participation in an educational setting for visually impaired and blind adolescents? A systematic review

**DOI:** 10.1186/s12886-023-03267-8

**Published:** 2024-01-04

**Authors:** Nina Milde, Diana Chabané Schmidt, Anders Larsen, Line Kessel

**Affiliations:** 1 Department of Ophthalmology, Copenhagen University Hospital, Rigshospitalet - Glostrup, Glostrup, Denmark; 2 UCSF - Center for Health Research, Copenhagen University Hospital - Rigshospitalet, Copenhagen, Denmark; 3 Department of Clinical Medicine, University of Copenhagen, Copenhagen, Denmark

**Keywords:** Visual impairment, Blindness, Adolescents, Rehabilitation, Education, Improved participation

## Abstract

**Background:**

Visually impaired and blind adolescents fare poorly in educational attainment compared to adolescents without vision impairment. Rehabilitation holds the potential to compensate for the hindrances that the impairment causes. Many rehabilitation initiatives exist. However, the efficacy of these initiatives remains uncertain. This systematic review assessed which rehabilitation initiatives improve participation in an educational setting for visually impaired and blind adolescents.

**Methods:**

PubMed, Embase, Scopus, Cinahl, and Cochrane library databases were searched. Only primary studies as randomized controlled trial (parallel group or crossover), cohort studies, case-control studies, qualitative studies, and case-studies were included. Data on the study characteristics, visual impairment, type of intervention, research question, main findings, and implications for practice were extracted from the papers. Critical appraisal was performed using the Critical Appraisal Checklist for Qualitative Research and the Checklist for Quasi-Experimental Studies both from the Joanna Briggs Institute. The data extraction and the critical appraisal were performed independently by two reviewers.

**Results:**

A total of 10 studies with visually impaired and blind adolescents were considered eligible, from an original search result of 3210 studies. In the thematic analysis we identified a heightened focus on different means for studying by making the curriculum content more accessible by applying different audio, tactile, or electronic devices (n = 8). A minor focus in the identified studies (n = 2) was placed on the impact of support from the environment on the development of literacy, for example the support from teachers or parents. Outcome parameters representing more diverse rehabilitation initiatives have not been adequately investigated in the literature. The scientific evidence that we identified was based on few publications with contradictory results and some studies were of questionable quality, limiting the applicability of their findings.

**Conclusions:**

Overall, the review identified a gap in the evidence regarding rehabilitation initiatives for visually impaired and blind adolescents that enables participation in an educational setting. The overall quality assessment of the 10 studies identified several risks of bias, for which reason the current scientific evidence does not qualify as a basis for decision making, leaving the adolescents in a heightened risk to fall even further behind in the educational system. Further high quality randomized controlled trials are required to establish high-quality evidence.

**Supplementary Information:**

The online version contains supplementary material available at 10.1186/s12886-023-03267-8.

## Introduction

Studies show that visually impaired (VI) and blind adolescents and adults in general fare poorly in educational attainment compared to their peers without vision impairment [1, 2]. This also affects the possibility for employment creating a significant employment gap between VI and blind adults compared to people without impairments [3–5]. A recent study has shown that when adolescents with VI or blindness caused by retinal disease move into adulthood, a negative socio-economic gradient is observed in Denmark [6]. Among other things, this is reflected in an elevated proportion of VI and blind adolescents with primary education as the highest attained education compared to a group without vision impairment. This discrepancy exists despite grade mark points from primary education were comparable with fellow peers, suggesting that the difference was not explained by intellectual differences between the groups [6]. In accordance with the Convention on the Rights of Persons with Disabilities [7] State Parties to the convention, are obliged to ensure and promote the full realization of *“(…) all human rights and fundamental freedoms for all persons with disabilities”* [7]. Rehabilitation can be a means for ensuring this obligation, and a tool for enabling the adolescents to complete a qualifying educational program.

Current recommendations on rehabilitation in relation to the participation in an educational setting suggest special counselling to the school delivered by a vision therapist or an assistive teacher for children and adolescents with VI or blindness. Counselling should focus on assistive devices, special educational needs, braille instruction, social and emotional development, psychological counselling, peer support, appropriate spectacles, mobility, orientation, and accessibility [8–10]. However, the rehabilitation initiatives for adolescents with VI or blindness is based on expert opinions and does not give answer to the expected effect on the different rehabilitation initiatives in relation to participation in an educational setting.

The aim of this systematic review was to identify primary studies and to evaluate which rehabilitation initiatives effectively improve participation in an educational setting for visually impaired and blind adolescents.

## Methods

We performed a systematic review with a focus on rehabilitation initiatives towards VI and blind adolescents. We were interested in identifying studies addressing rehabilitation initiatives in relation to an educational setting for VI and blind adolescents.

The article is presented in accordance with the PRISMA 2020 statement and checklist [11] (Additional file 1) and consult the ENTREQ reporting guidelines [12] for the presentation and synthesis of qualitative data.

The protocol for the review was registered in the PROSPERO database (reg. no. CRD42023430930). According to Danish Law institutional review board approval was not relevant for a systematic review.

### Eligibility criteria

The systematic review was based on a collection and synthesis of existing, primary studies that answered our research question. The primary studies were defined as randomized controlled trial (parallel group or crossover), cohort studies, case-control studies, cross-sectional studies, qualitative studies, and case-studies were included. We did not consider review articles, editorials, conference publications, books, or letters. Studies were eligible for inclusion in the systematic review if they focused on adolescents aged 12 to 17 years with VI or blindness. Studies based on adolescents with VI or blindness and severe comorbidities were excluded as we wanted to focus on the effect of visual impairment in itself. Definitions of VI and blindness followed the World Health Organization definition [13]: moderate VI was visual acuity worse than 6/18 to 6/60, severe – visual acuity worse than 6/60 to 3/60 and blindness – visual acuity worse than 3/60 or visual field < 20 degrees on the better seeing eye. We included studies targeted at interventions aimed at improving participation in an educational setting for VI or blind adolescents. Therefore, we were not interested in studies that investigated the best strategy to learn an assistive technology but focused on the effect that the assistive technology could have on an adolescent’s possibility to participate in an educational setting. We included studies conducted in western high-income countries. We only included full text studies disseminated in English, Danish, Swedish, or Norwegian. Studies conducted prior to year 2000 were excluded due to the increased development within the field of assistive technology devices in the latest decades.

### Information sources

We searched the following literature databases: PubMed, Embase, Scopus, Cinahl, and Cochrane library. The search strategy was developed in accordance with the PICO framework, and in collaboration with a trained information specialist (AL) (Additional file 2). The databases were searched from May 15th, 2023, to May 22nd, 2023. The searches were performed independently by one reviewer (NM) and an information specialist, to enable comparison of the number of results from every search in each of the five databases. The searches resulted in an identical number of search results for both the reviewer (NM) and the information specialist in each of the five databases.

### Selection process

All identified studies were exported to EndNote and thereafter imported to Covidence. Covidence is an online software for managing and streamlining systematic reviews. Covidence ensures transparency in the screening process, keeps track on the references, and generates the PRISMA flow diagram (www.covidence.org).

Two reviewers (NM and DS) independently performed the initial title and abstract screening and excluded obviously irrelevant studies or duplicates. The full text screening was conducted independently by the same two reviewers. Disagreement in both the initial title and abstract screening, and the full text screening were discussed between the two reviewers (NM and DS) and resolved. If agreement was not reached, a third reviewer (LK) was consulted for the final decision.

### Data collection and risk of bias of individual studies

To enable the data collection process an excel sheet for data extraction was developed by one reviewer (NM) and consulted with a second reviewer (DS). We extracted data on study characteristics (identifier of the study, type of study, participant characteristics (number of participants, age, and sex), visual impairment, type of intervention, research question, main findings, and implications for practice) (Additional file 3). If studies reported data on a broader age group than 12 to 17 years, we focused our data extraction on the study participants who were between 12 and 17 years of age. We incorporated a critical appraisal/risk of bias assessment. For qualitative studies the Critical Appraisal Checklist for Qualitative Research from the Joanna Briggs Institute (JBI) was applied [14]. One study [15] was a quasi-experimental study, for which reason the Checklist for Quasi-Experimental Studies (non-randomized experimental studies) also from JBI, was applied [16] (Tables 1 and 2).

Two reviewers (NM and DS) independently performed the data extraction and evaluated risk of bias. Disagreement was resolved through discussion. If agreement was not reached a third reviewer (LK) was consulted for final decision.

Due to the heterogeneity of the identified studies, it was not possible to perform a meta-analysis.

## Results

### Study selection

**Fig. 1:**
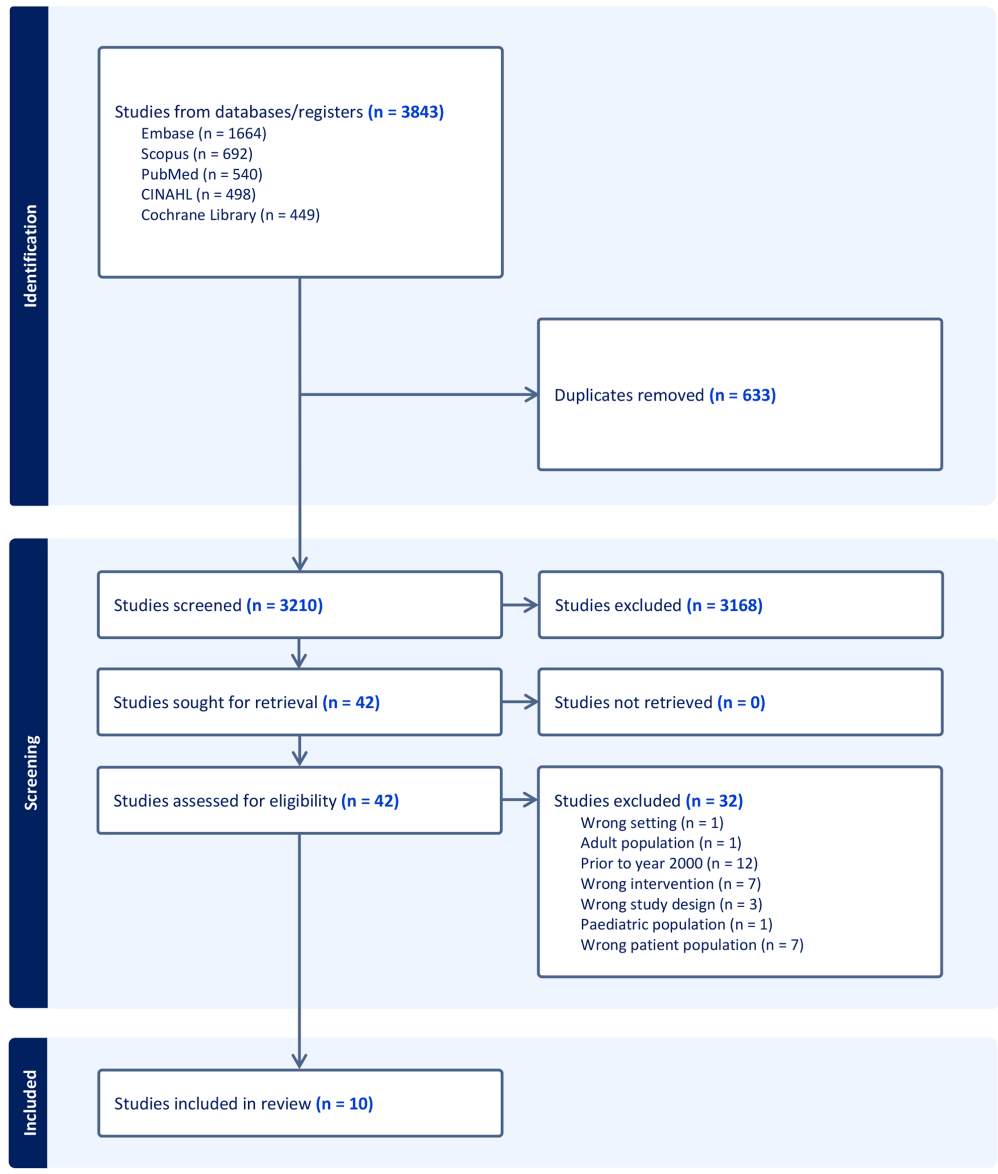
PRISMA flow diagram illustrating the study selection process

A total of 3843 studies were identified across the five literature databases. Covidence identified and removed a total of 633 duplicates, leaving 3210 studies left to screen. In the title and abstract screening process we excluded a total of 3168 studies, leaving 42 studies to be reviewed in full text. Finally, we excluded 32 studies as they did not fulfill our inclusion criteria. A total of 10 studies were considered eligible and included in the review. The selection process is outlined in the PRISMA flow diagram (Fig. 1).

The 10 studies collectively summarized data on 224 adolescents, 13 parents, 27 classroom- and assistive teachers, 10 teachers for visually impaired, and eight teachers from a national resource center for visually impaired. Of the 224 adolescents we estimated that 153 adolescents fulfilled the inclusion criteria: age 12 to 17 years, VI or blind without comorbidities. Out of the 153 adolescents, 95 had moderate to severe visual impairment, and 58 were blind.

Nine studies were qualitative, and one was a quasi-experimental study. Studies were conducted in The United States (n = 7), Norway (n = 1), Greece (n = 1), and in France (n = 1). Studies were reviewed qualitatively, and a thematic analysis was applied. Following an inductive approach to the thematic analysis three main themes emerged that are presented below.

The majority of studies focused on a medium for studying (n = 8). Out of the eight studies, four studies concentrated on more traditional media for studying, e.g. braille, large print, and simple supported eText [17–20], whereas the rest tested the efficacy of newly developed products for the delivery of learning content [15, 21–23]. Therefore, the studies are divided into two groups: *‘Medium for access to the core curriculum – Traditional means’* and *‘Medium for access to the core curriculum – New products’*.

**Table 1:** Critical appraisal for qualitative research

Study	Q1	Q2	Q3	Q4	Q5	Q6	Q7	Q8	Q9	Q10
Argyropoulos et al. (2019)	U	Y	Y	Y	Y	N	N	Y	Y	Y
Bouck and Weng (2014)	U	Y	Y	Y	Y	N	N	Y	N	Y
Chang and Schaller (2002)	U	Y	Y	Y	Y	N	N	Y	N	Y
Emerson and Anderson (2018)	U	Y	Y	Y	Y	N	N	Y	N	Y
Hahn et al. (2019)	U	Y	Y	Y	Y	N	N	Y	N	Y
Landau et al. (2013)	U	Y	Y	Y	Y	N	N	Y	N	Y
Rosenblum and Herzberg (2015)	U	Y	Y	Y	Y	N	N	Y	Y	Y
Rovira and Gapenne (2009)	U	Y	Y	Y	Y	N	N	U	N	Y
Vik (2008)	Y	Y	Y	Y	Y	N	N	U	Y	Y
% Yes responses	11	100	100	100	100	0	0	77	33	100

*Note*: Y = yes, indicates a clear statement appears in the paper which directly answers the question; N = no, indicates that the question has not been answered in the paper; U = unclear, indicates there is no clear statement in the paper that answers the question or there is ambiguous information presented in the paper. Criteria for the critical appraisal of qualitative evidence:

Q1: Is there congruity between the stated philosophical perspective and the research methodology?

Q2: Is there congruity between the research methodology and the research question or objectives?

Q3: Is there congruity between the research methodology and the methods used to collect data?

Q4: Is there congruity between the research methodology and the representation and analysis of data?

Q5: Is there congruity between the research methodology and the interpretation of results?

Q6: Is there a statement locating the researcher culturally or theoretically?

Q7: Is the influence of the researcher on the research, and vice versa, addressed?

Q8: Are participants, and their voices, adequately represented?

Q9: Is the research ethical according to current criteria or, for recent studies, is there evidence of ethical approval by an appropriate body?

Q10: Do the conclusions drawn in the research report flow from the analysis, or interpretation, of the data?

Abbreviations: N, no; U, unclear; Y, yes

**Table 2:** Critical appraisal for quasi-experimental studies

Study	Q1	Q2	Q3	Q4	Q5	Q6	Q7	Q8	Q9
Murphy and Darrah (2015)	Y	Y	U	N	N	Y	Y	Y	Y

*Note*: Y = yes, indicates a clear statement appears in the paper which directly answers the question; N = no, indicates that the question has not been answered in the paper; U = unclear, indicates there is no clear statement in the paper that answers the question or there is ambiguous information presented in the paper. Criteria for the critical appraisal of quasi-experimental studies:

Q1: Is it clear in the study what is the ‘cause’ and what is the ‘effect’ (i.e. there is no confusion about which variable comes first)?

Q2: Were the participants included in any comparisons similar?

Q3: Were the participants included in any comparisons receiving similar treatment/care, other than the exposure or intervention of interest?

Q4: Was there a control group?

Q5: Were there multiple measurements of the outcome both pre and post the intervention/exposure?

Q6: Was follow up complete and if not, were differences between groups in terms of their follow up adequately described and analyzed?

Q7: Were the outcomes of participants included in any comparisons measured in the same way?

Q8: Were outcomes measured in a reliable way?

Q9: Was appropriate statistical analysis used?

Abbreviations: N, no; U, unclear; Y, yes

### Medium for access to the core curriculum – traditional means

Several studies explored which medium effectively enabled the adolescents to participate in an educational setting. Argyropoulos et al. [17] examined the preferences and choices of VI and blind students’ literacy medium for studying. They found that 40% of adolescents between 12 and 15 years of age preferred braille, 59% preferred large print, and 1% preferred screen reading software. It’s worth noting that out of the 40% who preferred braille the vast majority were blind (96%). Likewise, out of the 59% who preferred large print only VI adolescents stated this as their preferred medium for studying. However, a total of 83% adolescents self-reported that they performed best by listening (94% of VI, 62% of blind), 16% self-reported that their best performance medium was braille or large print (35% blind, 6% VI), and only 1% (4% blind) preferred a combination of media.

The study by Bouck & Weng focused on the use of algebra supported eText. Supported eText is textual materials presented electronically or digitally. Bouck & Weng tested the use of algebra supported eText versus the students traditional means of accessing a textbook (for example braille, large print, computer screen software) and found that both the VI and blind adolescents performed slightly better when using traditional means of access (32% for eText – 38% for traditional means of access) [18].

Emerson & Anderson wanted to identify which level of oral description was most useful for VI and blind adolescents to understand specific types of images in math problems, where use of the image is necessary for completing the problem [19]. They created digital files containing math problems with examples and surrounding textual materials, making all elements (words, math expressions, and descriptions of images) readable by JAWS, a computer screen reader program. They found that an extended level of oral description resulted in an average of 28.6% correct answers compared to 15.1% in the control condition (no description), thereby enhancing the accessibility for specific types of images in math problems. However, even though an extended level of description improved the outcome, the result still reflected the inherent challenge of making math content accessible for visually impaired.

Another study by Rosenblum and Herzberg [20], solely involving braille readers, explored the adolescents’ opinions regarding which qualities compromised a good tactile graph, and whether the adolescents had inputs into the preparation of the tactile graphics. They found that the adolescents reported events where they were not provided with materials in braille, or that the materials were of a poor quality. When such situations arose the adolescents mainly turned to their peers or the assistive teacher (paraeducator). The adolescents described the need for different textures for different parts, lines that were distinct from each other, clear braille, a logical key, and being free of clutter, as elements that compromised a good tactile graph.

### Medium for access to the core curriculum – new products

Four studies focused on whether newly developed assistive technology devices could make learning content more accessible, especially content related to science, technology, engineering, and mathematics (STEM). Landau et al. [21] compared the effect of using an audio-tactile computer peripheral device, the Talking Tactile Tablet to the preferred current method of accommodation (braille, items read aloud, magnification) for administering multiple-choice math tests. Landau et al. showed that VI and blind adolescents performed better on five out of eight items when using the Talking Tactile Tablet and performed the same on the remaining three items compared to the use of current means.

Rovira and Gapenne [22] studied three blind adolescent’s categorization strategies used in 2-D geometric assignments performing identical tasks either with traditional material (thermoformed paper) or with a computerized platform called Tactos. The Tactos Platform consists of a graphics tablet and its pen, a computer, and a tactile stimulator. Tactos provides the possibility to explore a line or a graph by moving the pen on the graphic tablet. The adolescents were introduced to the Tactos devices, and followed for 20 h, during which time they became familiar with the Tactos device. Rovira and Gapenne showed in several trials that Tactos did not seem to lead to a radically different style of task management, which may indicate that Tactos can be considered as a complementary system in learning to read two-dimensional graphic objects, particularly in computerized environments [22].

Hahn et al. explored the effectiveness of multimodal touch screen tablet devices in conveying STEM graphics via vibrations and sounds [23]. They found that both the VI and blind adolescents were slightly more accurate when answering questions regarding an embossed graphic as opposed to a tablet graphic. However, their study design only included a brief introduction and training period to the tablet and application, whereas the relative equivalence in performance were interpretated as promising given the unfamiliarity of the new medium.

Murphy and Darrah found that the use of haptics-based apps promoted learning of content from national science and mathematics standards for VI and blind adolescents [15]. The haptics-based apps combine touch-based vibration, audio, and high contrast graphics. The testing of six different apps (Exploring the Atom, Gravity on the Planets, Surface Area of a Cube, Exploring the Plant Cell, Blood Cells and Circulatory System) showed an improvement of the score compared to the pre-test score (p < 0.001).

### The impact of support from the environment on the development of literacy

Two studies focused on how supportive factors in the adolescents’ environment could have an impact on the development of their literacy. Chang and Schaller [24] focused on how adolescents with VI and blindness perceived the support from their teachers. Through their interviews with the adolescents, they found that both emotional and formal support were highly valued. The adolescents wanted to be treated as individuals and enjoyed when teachers connected with them beyond what one would normally expect. On the other hand, several adolescents appreciated when their teachers regarded them just as capable as their sighted peers. The adolescents felt their learning was enhanced when teachers described a process in detail or gave sufficient instruction and support in the use of assistive technology.

Vik [25] investigated through interviews how the adolescents’ individual competencies, teacher and parent competence, and resources in the surrounding environment in different ways influenced the adolescents’ development of literacy. Vik found that an early intervention had a positive influence on the development of literacy, given that the VI was congenital or had emerged in infancy. If this was the case, parents reported that their child was evaluated by an interdisciplinary team at a National Resource Center for the Visually Impaired. Given that the recommendations from the National Resource Center for the Visually Impaired were followed, the child experienced to be tactually and visually stimulated at preschool and introduced to braille in first grade. Vik also highlighted that a stimulating environment for braille and print was established through availability of sufficient reading devices and collaboration in the surrounding environment, which positively affected the development of literacy.

## Discussion

In this systematic review based on 10 studies on rehabilitation initiatives that enables VI and blind adolescents to participate in an educational setting, we identified eight studies that concentrated on media for studying and two studies that focused on whether supportive factors in the adolescents’ environment could have an impact on the development of their literacy. Therefore, it seems that research is mainly focused on assistive devices/technology, and what effect these devices can have especially in relation to STEM subjects.

The identified studies highlighted an inherent inaccessibility in STEM related subjects. Four studies were concerned with the development of new assistive technology devices that make learning content in STEM more tactile and haptic. However, only the study concerning haptics-based apps by Murphy & Darrah and the study from Landau et al. concerning the Talking Tactile Tablet showed a positive effect in the adolescents’ performance when using newly developed assistive technology [15, 21]. Regarding the risk of bias in these studies, the study from Murphy and Darrah was robustly constructed and did not represent any considerable risk of bias (Table 2). The study from Landau et al. holds some risk of bias (Table 1). The other two studies [22, 23] did not find any improvement, on the contrary the adolescents performed slightly better when using their traditional medium for studying. The two studies differed in study design concerning introduction and training to the new medium. Rovira et al. had a training period of 20 h, and Hahn et al. had a training period of maximum 30 min. Both studies included participants who were skilled braille readers. Therefore, two conclusions can be drawn; training in the use of a new medium does not seem to lead to an elevated level of performance, and that the medium does not seem to impair the performance.

### Limitations

We are aware that this systematic review has some limitations. Our inclusion criteria concerning age, visual- and health status, and origin of the study most likely affected the number of identified studies. Regarding the age criteria, we estimated that the transition that adolescents must overcome are so special that it would be hard to make comparisons with initiatives tested or developed for younger children or adults with VI or blindness.

Likewise, we found it necessary to focus solely on the adolescents with isolated VI or blindness, in order to identify evidence on what compensates best for the loss of visual function rather than complex, multi-functional impairment. Lastly, the construct of health systems varies substantially between nations and cultures, and we wanted data from high income countries with comparable welfare systems.

Given the above-mentioned inclusion criteria the systematic review ended up being based on 153 individuals. This is a relatively small amount, considering the fact that there are an estimated 1.4 million blind children in the world [26]. Thus, it seems that rehabilitation initiatives of VI or blind adolescents in an educational setting is an underprioritized area of research.

### Methodological aspects

At present it remains a challenge to determine which rehabilitation initiatives that effectively improve VI and blind adolescents’ participation in an educational setting. To obtain an adequate level of evidence, we need to consider several methodological aspects: First, the population of adolescents with isolated VI or blindness is relatively small, and most countries do not have any systematic registration of this population.

Second, the highest level of scientific evidence is derived from trials using a randomized, controlled, double-blinded clinical trial design. In our review we have not identified one such trial, most likely because of the questions asked within this area of interest hardly can be investigated except in qualitative studies. Thomas et al. [27] sought to assess the effect of electronic assistive technologies on reading, educational outcomes, and quality of life in children and young people with low vision in a Cochrane Review including searches in ten databases. As they only wanted to include randomized controlled trials and quasi-RCTs they did not find any studies and concluded that high-quality evidence about assistive technology for children and young people are lacking. An aspect to take into consideration is the ethical problem that arises when withholding potential effective interventions from a control group, as must be done in a randomized controlled trial. In this review nine out of ten studies used a qualitative method, and one study was based on a quasi-experimental design. Most of the studies were constructed with congruity between methodology, research question, data collection, and analysis of the data and results (Tables 1 and 2). However, none of the nine qualitative studies stated their culturally or theoretical standpoint, or how they might influence the research, and only 33% of the studies stated evidence of ethical approval or argued why a formal approval wasn’t necessary. In qualitative research this could create a considerable risk of bias, as the researchers in qualitative research are in a heightened risk of impacting the outcome of the research. The included studies did not apply a homogenous way of evaluating their results: Three studies evaluated their research solely by using surveys/interviews, five studies used a combination of testing and follow-up interviews with participants, and two studies evaluated solely by testing. This diversity in methodological evaluation affects the quality of evidence and can be a hinderance concerning making comparisons. Furthermore, the heterogeneity of the study population (age of onset, severity of vision loss etc.) constitutes another methodological aspect that must be taken into consideration.

Third, two studies concerned with investigating whether the support from the environment had an impact in the development of literacy, concluded that this was very dependent on individual factors such as parents’ involvement, perceived support in school (the quality and expression), and accessibility of assistive devices and instruction hereof. Intelligence, social and cultural background might be other individual factors that influence the possibility for developing literacy. However, when studies conclude that the effect of initiatives solely depends on individual actions and preferences is it not possible to implement general recommendations.

The listed methodological aspects are a source of bias and remind us that a systematic review can only provide conclusions as good as the studies included.

## Conclusion

In conclusion, we find that there is a gap in the evidence regarding rehabilitation initiatives for VI and blind adolescents that enables participation in an educational setting. The scientific evidence that we managed to identify is based on few publications with contradictory results and some studies were of questionable quality. Studies that focus on more diverse aspects of rehabilitation are missing. This is problematic as the scientific evidence clearly states that the adolescents fall behind in the educational system.

Rehabilitation for adolescents with VI or blindness is a highly specialized and diverse area, and scientific evidence is needed to identify which rehabilitation initiatives are more useful. Such evidence would help and guide teachers and healthcare professionals to choose the most appropriate techniques, as well as guiding decisionmakers in the allocation of personal, institutional, and public resources. All with the purpose of enabling the adolescents to participate in an educational setting.

Therefore, the evidence identified in this systematic review does not qualify as a basis for decision making for which reason further attention is needed.

Likewise, another important question has emerged. Given that some of the rehabilitation initiatives have a positive effect on the participation in an educational setting, making the education more accessible, which long-term effects can be detected in relation to obtain a qualifying education or an independent way of life?

### Electronic supplementary material

Below is the link to the electronic supplementary material.

Additional file 1. PRISMA checklist.

Additional file 2. Search strategies.

Additional file 3. Data extraction sheet.

## Data Availability

The data used are directly extracted from the studies included in this systematic review, which are cited in the tables and in the text.

## Abbreviations

JAWS: Job Access With Speech ++
JBI: Joanna Briggs Institute
STEM: Science, technology, engineering, and mathematics
VI: Visually impaired
