# Supplementary material for: Which rehabilitation initiatives can effectively improve participation in an educational setting for visually impaired and blind adolescents? A systematic review
Source: BMC Ophthalmol. 2024 Jan 4;24:10. doi: 10.1186/s12886-023-03267-8 (PMC10768278; doi:10.1186/s12886-023-03267-8)
Supplement: Supplementary file 2 — Additional file 2. Search strategies. [file 12886_2023_3267_MOESM2_ESM.docx]

**Title: Additional file 2. Search strategies.**

**Legend: Search strategies for PubMed, Embase, Scopus, CINAHL, and Cochrane Library.**

**PubMed May 2023**

**P** #1 Adolescent[mh] OR

adolescen*[tiab] OR

teen*[tiab] OR

youth[tiab] OR

youngster*[tiab] OR

boy[tiab] OR

boyhood[tiab] OR

girl*[tiab] OR

girlhood[tiab]

#2 Eye Diseases[mh] OR

Vision Disorders[mh] OR

Visually Impaired Persons[mh] OR

blind*[ti] OR

blind*[ot] OR

"eye disease*"[tiab] OR

"eye disorder*"[tiab] OR

"impaired vision"[tiab] OR

"low vision"[tiab] OR

"visually impair*"[tiab]

**I** #3 Optometry[mh] OR

Rehabilitation[mh] OR

Rehabilitation[sh] OR

Audiovisual Aids[mh] OR

braille[tiab] OR

optometry[tiab] OR

"orientation and mobility"[tiab] OR

habilitation[tiab] OR

rehabilitation[tiab] OR

"visual aid*"[tiab] OR

Self care [mh] OR

Self care[tiab] OR

Self help[tiab] OR

Self management[tiab]

**C** -- --

**O** #4 Education[mh] OR

Education[sh] OR

education*[tiab] OR

school*[tiab] OR

schooling[tiab] OR

"high school*"[tiab] OR

"secondary school*"[tiab] OR

"secondary education*"[tiab] OR

schoolchild*[tiab]

#1 AND #2 AND #3 AND #4 = 540

**Embase May 2023**

**P** #1 exp adolescent/ OR

adolescent*.mp OR

teen*.mp OR

youth.mp OR

youngster*.mp OR

exp boy/ OR

boy*.mp OR

boyhood.mp OR

exp girl/ OR
girl*.mp OR

girlhood.mp

#2 exp eye disease/ OR

exp vision disorder/ OR

exp visually impaired person/ OR

blind*mp OR

"eye disease*".mp OR

"eye disorder*".mp OR

"impaired vision".mp OR

exp low vision/ OR

"low vision".mp OR

"visually impair*".mp

**I** #3 exp optometry/ OR

exp rehabilitation/ OR

exp audiovisual aids/ OR

exp braille/ OR

braille.mp OR

optometry.mp OR

"orientation and mobility".mp OR

Habilitation.mp OR

Rehabilitation.mp OR

"visual aid*".mp OR

exp self care/ OR

self care.mp OR

exp self help/ OR

self help.mp OR

self management.mp

**C** -- --

**O** #4 exp education/ OR

education*.mp OR

exp school/ OR

school*.mp OR

schooling.mp OR

exp high school/ OR

"high school*".mp OR

"secondary school*".mp OR

"secondary education*".mp OR

exp school child/ OR

school child*.mp

#1 AND #2 AND #3 AND #4 = 1664

**Scopus May 2023**

**P** #1 TITLE-ABS-KEY (adolescen*) OR

TITLE-ABS-KEY (teen*) OR

TITLE-ABS-KEY (youth) OR

TITLE-ABS-KEY (youngster*) OR

TITLE-ABS-KEY (boy*) OR

TITLE-ABS-KEY (boyhood) OR

TITLE-ABS-KEY (girl*) OR

TITLE-ABS-KEY (girlhood)

#2 TITLE-ABS-KEY (blind*) OR

TITLE-ABS-KEY ("eye disease*") OR

TITLE-ABS-KEY ("eye disorder*") OR

TITLE-ABS-KEY ("impaired vision") OR

TITLE-ABS-KEY ("low vision") OR

TITLE-ABS-KEY ("visually impair*")

**I** #3 TITLE-ABS-KEY ("audiovisual aids") OR

TITLE-ABS-KEY (braille) OR

TITLE-ABS-KEY (optometry) OR

TITLE-ABS-KEY ("orientation and mobility") OR

TITLE-ABS-KEY (habilitation) OR

TITLE-ABS-KEY (rehabilitation) OR

TITLE-ABS-KEY ("visual aid*") OR

TITLE-ABS-KEY ("self care") OR

TITLE-ABS-KEY ("self help") OR

TITLE-ABS-KEY ("self management")

**C -- --**

**O** #4 TITLE-ABS-KEY (education*) OR

TITLE-ABS-KEY (school*) OR

TITLE-ABS-KEY (schooling) OR

TITLE-ABS-KEY ("high school*") OR

TITLE-ABS-KEY ("secondary school*") OR

TITLE-ABS-KEY ("secondary education*") OR

TITLE-ABS-KEY (schoolchild*) OR

TITLE-ABS-KEY ("school child*")

#1 AND #2 AND #3 AND #4 = 692

**CINAHL May 2023**

**P** S1 MH "Adolescence+" OR

adolescen* OR

teen* OR

youth OR

youngster* OR
boy* OR
boyhood OR

girl* OR

girlhood

S2 MH "Eye Diseases+" OR

MH "Vision Disorders+" OR

Visually impaired persons* OR

TI blind* OR

SU blind* OR

(eye N2 disease*) OR

(eye N2 disorder*) OR

(impaired N0 vision) OR

"low vision" OR

(visually N0 impair*)

**I** S3 MH Optometry OR

MH "Rehabilitation+" OR

Braille OR
 MH Self care OR
 MH Self-management OR

braille OR

optometry OR

"orientation and mobility" OR

habilitation OR

rehabilitation OR

"visual aid*" OR

Self care OR

Self help OR

Self-management

**C** -- --

**O** S4 MH Education+ OR
 MH "Secondary school+" OR

MH "Schools, Secondary" OR

education* OR

schooling OR
 "high school*" OR
 "secondary education*" OR
 schoolchild*

#1 AND #2 AND #3 AND #4 = 498

**Cochrane Library May 2023**

**P** #1 Adolescent[mh] OR
 (adolescen*):ti,ab,kw OR
 (teen*):ti,ab,kw OR
 (youth):ti,ab,kw OR
 (youngster*):ti,ab,kw OR
 (boy*):ti,ab,kw OR
 (boyhood):ti,ab,kw OR
 (girl*):ti,ab,kw OR
 (girlhood):ti,ab,kw

#2 Eye Diseases[mh] OR

Vision Disorders[mh] OR

Visually Impaired Persons[mh] OR

Vision, low [mh] OR

(blind*):ti,ab,kw OR

("eye disease*"):ti,ab,kw OR

("eye disorder*"):ti,ab,kw OR

("impaired vision"):ti,ab,kw OR

("low vision"):ti,ab,kw OR

("visually impair*"):ti,ab,kw

**I** #3 Optometry[mh] OR

Rehabilitation[mh] OR

(rehabilitation):ti,ab,kw OR

Audiovisual Aids[mh] OR

("braille"):ti,ab,kw OR

(optometry):ti,ab,kw OR

("orientation and mobility"):ti,ab,kw OR

(habilitation):ti,ab,kw OR

("visual aid*"):ti,ab,kw OR

Self care[mh] OR

("self care"):ti,ab,kw OR

(self-help):ti,ab,kw OR

Self-management[mh] OR

Self-management:ti,ab,kw

**C** -- --

**O** #4 Education[mh] OR

(education*):ti,ab,kw OR

Schools[mh] OR

(schooling):ti,ab,kw OR

(high school*):ti,ab,kw OR

"secondary school*"ti,ab,kw OR

("secondary education*"):ti,ab,kw OR

(schoolchild*):ti,ab,kw OR

(school child):ti,ab,kw

#1 AND #2 AND #3 AND #4= 449
